# Beer Aroma Compounds: Key Odorants, Off-Flavour Compounds and Improvement Proposals

**DOI:** 10.3390/foods14244287

**Published:** 2025-12-12

**Authors:** Corina Maria Șutea, Elena Mudura, Carmen Rodica Pop, Liana Claudia Salanță, Anca Corina Fărcaș, Petruț Cristian Balaș, Emese Gal, Elisabeta-Irina Geană, Haifeng Zhao, Teodora Emilia Coldea

**Affiliations:** 1 Department of Food Engineering, Faculty of Food Science and Technology, University of Agricultural Sciences and Veterinary Medicine Cluj-Napoca, 400372 Cluj-Napoca, Romania; corina.sutea@usamvcluj.ro (C.M.Ș.); elena.mudura@usamvcluj.ro (E.M.); cristian.balas@usamvcluj.ro (P.C.B.); 2 Department of Food Science, Faculty of Food Science and Technology, University of Agricultural Sciences and Veterinary Medicine Cluj-Napoca, 400372 Cluj-Napoca, Romania; carmen-rodica.pop@usamvcluj.ro (C.R.P.); liana.salanta@usamvcluj.ro (L.C.S.); anca.farcas@usamvcluj.ro (A.C.F.); 3 Faculty of Chemistry and Chemical Engineering, Babeș-Bolyai University, 11 Arany Janos Street, 400028 Cluj-Napoca, Romania; emese.gal@ubbcluj.ro; 4 National Research and Development Institute for Cryogenics and Isotopic Technologies, Uzinei Str. No. 4, 240050 Ramnicu Valcea, Romania; irina.geana@icsi.ro; 5 School of Food Science and Engineering, South China University of Technology, Guangzhou 510640, China; hfzhao@scut.edu.cn

**Keywords:** aroma, odorants, off-flavours, beer, quality control

## Abstract

Beer aroma is a critical quality attribute shaped by volatile compounds from malt, hops, and yeast metabolism; however, the appearance of off-flavours can compromise it. This review identifies and describes the main aroma-active compounds that define the beer’s sensory profile, emphasising both key odorants and undesirable notes, such as diacetyl, dimethyl sulphide, acetaldehyde, and light-struck thiols. Based on recent literature, a troubleshooting guide was developed to link specific odours with their particular chemical compounds, origin, and corrective actions. The findings highlight how the composition of raw materials, processing and storage conditions, treatments, fermentation management, and even packaging play an important role in flavour stability. By identifying the sources of common defects and offering practical solutions, this work provides brewers with strategies to enhance aroma control and improve overall beer quality.

## 1. Introduction

Beer, the most widely consumed fermented alcoholic beverage globally, is produced from a carbohydrate-rich wort that undergoes yeast-driven conversions, resulting in both alcohol and carbon dioxide [1]. Beer quality is determined by a combination of sensory attributes, such as aroma, appearance, taste, and texture, which together shape the overall sensory perception of the product [2]. For consumers, aroma is closely linked to emotional responses and memory [3]. Pleasant aromas can improve the drinking experience, whereas off-flavours might signal potential quality issues [4].

One consideration when discussing aroma compounds in beer is that each beer style can have distinct patterns of compound abundance, as well as compounds that are exclusive to certain styles [5]. The diversity of beer styles is extensive, making it challenging to list them all. However, one of the primary classification methods is based on the yeast strain used for fermentation, which typically categorises most beers as either lager or ale, depending on the yeast species employed: *Saccharomyces pastorianus* for lager and *Saccharomyces cerevisiae* for ales [6,7]. The yeast strain and the composition of the wort significantly influence the flavour and aroma of a beer [8,9]. Additional classification factors for beer styles include ingredients, processing techniques, packaging, marketing strategies, and the cultural context surrounding the beer [10]. Furthermore, beer can be categorised by original gravity, degree of attenuation, alcohol content, foam characteristics, acidity, colour, mouthfeel, flavour, and aroma, as well as physiological effects [11].

One of the key characteristics of any food product that is important to the consumer is the odour produced by odour-active compounds that exceed a certain odour detection threshold, also known as aroma value or odour activity value, making the volatile food constituents perceptible to the human nose [12,13]. The odour-active compounds are volatile, but not all volatile compounds present in food are in concentrations high enough to exceed the odour threshold; for example, out of approximately 8000 volatiles found in food products, only 5% are detectable by the human nose and influence the aroma [14].

**Figure 1:**
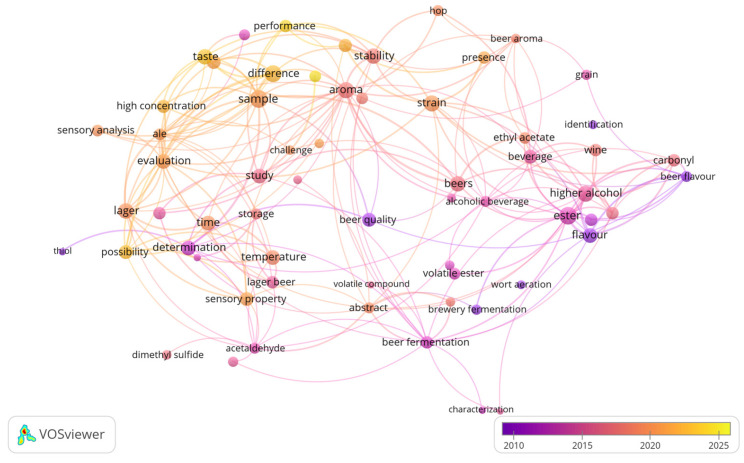
Network map of keyword co-occurrences coloured according to year.

Key odorants are volatile compounds in beer that significantly contribute to its sensory characteristics. Identifying key odorants and their effects will enable brewers to precisely adjust processes or practices to influence the development of these compounds positively. This review aims to identify and describe key aroma compounds found in various beer styles. It also seeks to develop a troubleshooting guide to recognise and address common off-flavours and their causes, providing practical solutions to improve beer quality. To visualise the research trends, a keyword co-occurrence network was generated using VOSviewer version 1.6.20 (Figure 1). The map displays the most frequently co-occurring keywords within the analysed literature and the relationship between words, while also providing an overview of the thematic evolution of flavour research over time.

## 2. Key Odours and Common Off-Flavours Found in Beers and Their Sources

Beer odours mainly originate from the natural chemical volatiles present in barley malt, hops, and yeast metabolism [1]. Off-flavours, on the other hand, are unpleasant odours or tastes caused by the deterioration of a food product [15]. This chapter concentrates on the key odours and off-flavours typically found in beer, offering detailed descriptions of the responsible odorants and their detection thresholds. Detection thresholds are widely accepted in flavour science as a key parameter for identifying odour-active compounds whose concentrations are high enough to be perceptible by the human nose and relevant for sensory quality control [16].

### 2.1. Apple

The odour of acetaldehyde is often described as similar to that of green apples or unripe apples, but at higher concentrations, it can impart an emulsion, paint-like odour to beer [17]. Acetaldehydes are a secondary product of alcoholic fermentation, formed during yeast metabolism of sugars through the action of pyruvate decarboxylase and alcohol dehydrogenase [18]. In most standard beers, acetaldehyde concentrations are generally low because yeast efficiently reduces acetaldehyde to ethanol during maturation. For lager beers, typical concentration levels of acetaldehyde usually do not exceed 0.01 mg/L [1]. While high concentrations are considered unpleasant and negatively affect beer quality, appropriate levels can contribute to a pleasant green apple aroma [19]. By contrast, much higher concentrations can occur in specific beer types or under certain processing conditions. For example, in alcohol-free beers, concentrations were reported at approximately 1.2 ± 0.05 mg/L, with the ortho-nasal odour detection threshold of 0.0458 mg/L [20]. Similarly, in Bavarian wheat beers, acetaldehyde concentrations ranged between 0.52 and 1.72 mg/L. The odour activity value (OAV), calculated by dividing the odourant concentration by its odour threshold, was reported as 21 and 69 for these beers [21]. These style-dependent differences arise from variations in yeast strains, fermentation conditions, and the extent of maturation [1]. In general, in the context of food odourants, odour thresholds for acetaldehydes, determined in water, range from 0.015 to 0.120 mg/L. The recognition threshold is typically cited as 0.063 mg/L, with the detection threshold at 0.025 mg/L [12].

### 2.2. Alcoholic

The most essential alcohols present in beer are ethanol and fusel alcohols, also known as higher alcohols. These include propanol, isoamyl alcohol, active amyl alcohol, isobutanol, and 2-phenylethyl alcohol [22]. An alcoholic odour is usually characterised as a strong and pungent smell, especially when higher alcohols are present in beer in quantities greater than 300 mg/L [23]. The higher alcohols can be classified into aliphatic (n-propanol, isoamyl alcohol, isobutanol), which contribute to the alcoholic aroma, and aromatic higher alcohols, represented by 2-phenylethyl alcohol, which imparts a positive, sweet, rose-like aroma in beers [24,25]. The production of higher alcohols is linked to the metabolism of amino acids, where, through the Ehrlich pathway (a catabolic pathway), amino acids undergo transamination to produce α-keto acids, which are later decarboxylated and then reduced to produce higher alcohols by the yeast [9,26]. The higher alcohols produced via this pathway include isoamyl alcohol, active amyl alcohol, isobutanol, and phenylethyl alcohol, which originate from the amino acids leucine, isoleucine, valine, and phenylalanine [24]. Higher alcohols can also be formed through the anabolic pathway, such as propanol, which is derived from threonine via oxidative deamination [24]. An unpleasant odour in higher alcohols is associated with amyl alcohol, which can significantly affect drinkability if its concentration increases [27]. Higher alcohols are more common in beers classified as high-alcohol varieties, such as Belgian Dubbels and Tripels, which emerged as a consequence of the Vandervelde Act. This legislation prohibited the sale of spirits in pubs throughout Belgium from 1918 until 1983 [28].

### 2.3. Boiled Potato

One of the Strecker aldehydes responsible for the potato odour is methional, which originates from the amino acid methionine, a compound usually reduced by yeast during fermentation [20]. According to Piornos et al. (2020), methional is present in alcohol-free beers produced using a cold-contact fermentation method at a concentration of 0.0854 mg/L [20], while in other studies on pilsner beer, the compound was found at a concentration of 0.0012 mg/L [29]. Generally, Strecker aldehydes like methional occur at lower concentrations in lager beers [20]. The orthonasal detection threshold for methional ranges from 0.0002 to 0.0018 mg/L [16].

### 2.4. Buttery

Diacetyl, also known as 2,3-butanedione, is a vicinal diketone that imparts a buttery or butterscotch aroma [30]. The diacetyl concentration in beer varies, ranging from 0.016 to 0.036 mg/L, depending on the beer style [31]. The odour threshold of diacetyl is very low for beer, ranging from 0.1 to 0.2 mg/L in lagers and 0.1 to 0.4 mg/L in ales. When the concentration exceeds this sensory threshold, the product will smell and taste spoiled [32]. There is also 2,3-pentanedione, another vicinal diketone, which can produce similar notes in beer, such as diacetyl, but is also described as more toffee-like, honey-like, or creamy, generally [30,33]. Diacetyl is produced by non-enzymatic oxidative decarboxylation of α-acetolactate, an intermediate in valine and leucine biosynthesis, whereas 2,3-pentanedione is formed by yeast from intermediates of isoleucine synthesis [34]. In some cases, for specific beer styles, such as ales, diacetyl is desired in small amounts to round out the flavour, but it can easily become excessive [35].

### 2.5. Caramel, Malty

The caramel and malty notes in beer generally arise from Maillard reactions, caramelisation, pyrolysis products, or are linked to Maillard-derived compounds [36,37]. The key chemical classes and compounds responsible for the caramel and malty aroma include: furans and furan derivatives (e.g., furfural, 5-methylfurfural, furfuryl alcohol) [38], heterocycles and pyrazines (e.g., pyrroles, pyridines, pyrazines) [39], and carbonyl compounds along with related Maillard reaction products (maltol, isomaltol, 4-hydroxy-2,5-dimethylfuran-3(2H)-one (HDMF, also called furaneol) [40]. Furfural, belonging to the aldehyde group, is found at concentrations between 2.6 and 7.77 mg/L, making it one of the most prevalent aldehydes in beers [41]. When present near the sensory threshold, HDMF imparts a pineapple aroma; however, depending on the wort’s original gravity and the yeast strain used, it can also add a sweet, caramel aroma to beer [42].

### 2.6. Cidery

The cidery character is usually linked to acetaldehydes, which are also characterised by a green apple aroma [35]. Some esters can also contribute sour apple notes, such as ethyl caproate, ethyl caprylate, or even ethyl acetate, when present in low concentrations [43]. In lager beer, the concentration of ethyl caproate at 0.05–0.3 mg/L is close to the detection threshold of 0.17–0.21 mg/L, while ethyl caprylate occurs at 0.04–0.53 mg/L, also near the detection threshold of 0.3–0.9 mg/L. In contrast, ethyl acetate is present at much higher levels, ranging from 8 to 32 mg/L, but its threshold level is elevated at 21 to 30 mg/L [44]. The cidery flavour can also result from a high proportion of simple sugars, such as glucose and sucrose, or the oxidation of acetaldehyde, which can produce acetic acid and contribute to the cidery taste [35].

### 2.7. Cooked Vegetable

Dimethyl sulphide is one of beer’s most studied volatile sulphur compounds, often described as having a sweetcorn, cooked vegetable, baked bean, or tinned tomato-like odour [45]. The level of dimethyl sulphide (DMS) in beer correlates with its precursor, S-methylmethionine (SMM), a non-protein amino acid synthesised during germination that is heat-labile [46]. A second source of DMS is dimethyl sulfoxide (DMSO), which results from the breakdown of SMM during malt curing [47]. Threshold values for DMS vary depending on the beer’s chemical composition; the literature reports ranges between 0.03 and 0.10 mg/L, determined by different sensory tests, with subthreshold levels positively influencing beer flavour and being desirable. However, levels above 0.10 mg/L can impart a vegetable-like or cabbage taste [46,48]. It is recommended to keep DMS and SMM levels below 0.10–0.12 mg/L at the start of fermentation [49]. Dimethyl sulphide is commonly present in lagers and contributes to their characteristic aroma; thus, a certain amount is desired [50].

### 2.8. Estery, Fruity

Isoamyl acetate is an aliphatic acetate ester characterised by a fruity, banana, sweet, fresh, and apple aroma [51]. In Hefeweizen beers, the banana-like flavour imparted by isoamyl acetate represents a key flavour attribute [52]. Ethyl hexanoate and ethyl octanoate are aliphatic ethyl esters; ethyl hexanoate contributes apple, fruity, and aniseed aromas, while ethyl octanoate offers sweet, fruity, and apple aromas [42]. A high concentration of ethyl octanoate can lead to a lager with an unpleasant, solvent-like smell [53]. Ethyl benzoate has a fruity aroma at lower concentrations, but it can produce a negative, harsh, solvent-like flavour at concentrations as low as 0.10 mg/L [53]. Phenylethyl acetate is an acetate ester that imparts rose, floral, fruity, sweet, and honey notes [51]. When present in high concentrations, phenylethyl acetate can produce a pungent solvent odour [53]. The detection thresholds for isoamyl acetate, ethyl hexanoate, ethyl octanoate, and phenylethyl acetate are 1.2, 0.21, 0.9, and 3.8 mg/L, respectively [54]. In beer, their concentrations range from 0.079 to 0.489 mg/L for isoamyl acetate, 0.081 to 0.411 mg/L for ethyl hexanoate, 0.04 to 0.53 mg/L for ethyl octanoate, and 0.1 to 0.73 mg/L for phenylethyl acetate [55]. Besides esters, some higher alcohols, thiols, lactones, furanones, and terpenoids can also contribute to the fruity character of a beer. Some more exotic fruit flavours originate from polyfunctional thiols found in hops rather than esters; the best-known examples are 3-sulfanyl-4-methyl-pentan-1-ol (exotic fruit, rhubarb, grapefruit, present in hop varieties Nelson Sauvin, Hallertau Blanc, Tomahawk), 4-methyl-4-sulfanyl-2-pentanone (exotic fruit, rhubarb, grapefruit, an extremely potent thiol with a sensory threshold of 1.5 × 10^−6^ mg/L, found in hop varieties such as Simcoe, Summit, Cascade), 3-sulfanylhexan-1-ol (passion fruit, present in the hop variety Tomahawk), and p-menthane-8-thiol (grapefruit; its oxidised derivative, p-menthane-8-thiol-2-one, can be an off-flavour in aged beer) [42]. For odours with descriptors such as peach and apricots, several lactones are responsible, namely γ-decalactone and δ-decalactone, which originate from malt, hops, and yeast metabolism [42].

### 2.9. Grassy

Most of the time, aldehydes are responsible for the grassy odour, and the presence of aldehydes above the threshold limit (10–20 mg/L) can cause grassy off-flavours [9]. In kilned malts, aldehydes are found in smaller amounts, but in green malt and green malt wort, they contribute to the grassy, green, pea-like, and beany taste and aroma [9]. Hexanal is an aldehyde that imparts a grassy, vinous, and aldehydic odour, with a threshold of 0.088–0.35 mg/L and a concentration of 0.0004–0.0007 mg/L in fresh beer, increasing to 0.0013–0.001.8 mg/L in aged beer [56].

### 2.10. Hoppy

The hoppy aroma in beer mainly originates from volatile compounds present in hops, especially essential oils, which constitute only about 0.5 to 3% of the hop cone’s dry matter [57]. These oils are complex mixtures of monoterpenes, sesquiterpenes, oxygenated compounds, and thiols [58], produced either by the hop plant itself or formed via oxidative and hydrolytic reactions occurring during brewing [57]. Monoterpenes and sesquiterpenes comprise 70–80% of the hop essential oil, depending on the variety, with detection limits of 0.35 mg/L for β-myrcene, 0.45 mg/L for α-humulene, and 0.23 mg/L for β-caryophyllene [58]. Among the prevalent terpenoids, myrcene is distinguished by its pungent, resinous, herbaceous, balsamic, and geranium-like aroma [59,60]; α-humulene imparts oily, green, woody, and balsamic scents; β-caryophyllene offers green, spicy, woody, and clove-like as well as turpentine notes [59]; and β-farnesene contributes woody, citrus, herbal, and sweet notes [61]. Other compounds contributing to the hoppy aroma include generally linalool (floral, citrusy) [57,59,62], geraniol (floral, rose, geranium), β-damascenone (cooked apple, tobacco, prunes) [57,59], β-citronellol, esters (notably ethyl 4-methylpentanoate and (Z)-4-decenoate), and organic acids (such as 2- and 3-methylbutanoic) [57].

### 2.11. Husky, Grainy

Grainy aromas can originate from isobutyraldehyde (2-methylpropanal) [63], a Strecker aldehyde formed from the amino acid valine [64]. Concentrations in fresh beer were reported to range from 0.0044 to 0.0065 mg/L, and in aged beer, they can reach 0.0668 mg/L [65]. There are also some volatile nitrogenous compounds, such as ethyl nicotinate and *o*-aminoacetophenone, associated with grainy flavours in beer at threshold values of 2 mg/L [66] and 0.005 mg/L, respectively [11]. Concentrations of ethyl nicotinate in beers after a month of storage ranged from 0.00286 to 0.02256 mg/L, and after six months of ageing, they increased to 0.021–0.1472 mg/L [67]. The husk and pericarp together account for about 7–14% of the grain, depending on the barley variety, size, and growing environment [68]. If the malt polyphenols from the husk are not extracted during brewing, they will impart a husky or grainy flavour [68].

### 2.12. Medicinal

Responsible for medicinal, antiseptic, and hospital odours are chlorophenols (e.g., 2,6-dichlorophenol) and bromophenols, which form when chlorine from water reacts with phenolic compounds naturally present in beer [45]. In other cases, trichlorophenol (TCP) can form from the reaction between plastic tubing and hypochlorite-based cleaning agents, resulting in a medicinal flavour [45]. Some consumers are sensitive to phenol compounds, while others are described as “taste-blind,” but overall TCP has been detected at concentrations as low as 0.001 mg/L [69].

### 2.13. Metallic

One of the main causes of the metallic, blood-like, rusty, coin-like, or tinny taste and odour in beer is the presence of iron in its most soluble form, ferrous (Fe^2+^), which can be found in water [70]. Besides iron, copper is also frequently described as having a metallic flavour [70,71]. The concentrations of iron and copper in beers vary widely depending on origin, ranging from 0.015 to 1.55 mg/L for Fe and 0.008 to 0.80 mg/L for Cu [72]. The flavour threshold for iron is reported as 0.05 mg/L [70]. When iron levels are too high, they can affect the quality of the final product and cause water to turn orange or rust-coloured, or lead to corrosion of stainless-steel piping, especially if combined with chlorides and sulphides [52]. Fe^2+^ is present if iron-containing water is not aerated; in aerated iron-containing water, this will contain ferric ion, Fe^3+^ [52]. Aqueous solutions containing Fe^3+^ do not have a metallic flavour [70].

### 2.14. Mouldy

Moulds are rare in beer, but if they are present in raw materials, they can cause the development of strong off-flavours and impact the quality of the malt, wort, and ultimately the beer [73]. The most common fungi found in barley fields include *Alternaria, Aureobasidium, Cladosporium*, *Epicoccum, Fusarium,* and *Helminthosporium*; in barley storage, *Aspergillus* and *Penicillium* [74]. Some compounds produced by mould or bacteria growth emit earthy, fungal, and musty odours even at very low detection thresholds, including geosmine, 2-methyl isoborneol, 1-octen-3-one, and chloroanisoles [75]. Geosmin is a sesquiterpene produced by various microorganisms such as bacteria, actinomycetes, algae, and moulds (*Penicillium*) [76]. In water, it has a detection threshold of 5 × 10^−6^ mg/L and an identification threshold of 1 × 10^−5^ mg/L [76]. A likely source of geosmin and isoborneol is contaminated water supplies with actinomycetes [77]. 1-octen-3-one is a degradation product from the reaction between Fe(II) and lipids, described as having a metallic or mushroom-like [70]. The compound haloanisoles, specifically 2,4,6-trichloroanisole (TCA), cause unpleasant odours of mould, wet cardboard, and damp cellars in beer when water or raw materials are contaminated. TCA is mainly produced by fungal species of the genera *Aspergillus* and *Penicillium* [76,78]. In wines, TCA is responsible for the off-flavour known as cork taint [78]. The detection limit for haloanisoles in beer and wine is between 1.29 × 10^−5^ and 2.08 × 10^−5^ mg/L [79]. In cases of microbial contamination in packaging, the chemical compound 2-ethyl fenchol may emerge, imparting earthy, soil, compost, or mouldy odours in beer. Its detection threshold in water and spoilage concentration in beer are both considered to be 0.005 mg/L [80].

### 2.15. Nutty

Furans derived from the Maillard reaction can be oxidised, and some have low odour thresholds, such as 2-acetylfuran, a typical ageing compound with sweet and nutty notes [45,81]. The odour threshold for 2-acetylfuran was reported to be 0.513 mg/L [56]. In lagers, the concentration ranged between 0.0049 and 0.0068 mg/L in fresh beers and between 0.0137 and 0.0157 mg/L in aged beers [56]. During the Maillard reaction, pyrroles (sweet, nutty, liquorice), pyrazines (cocoa, roasted nuts, peanut butter, coffee), and thiazoles (nutty, green, bean sprout) are also formed [45]. 2-acetylpyrrole has a nutty, sweet, ethereal flavour, but it can also produce mouldy, leathery, cherry, cherry liqueur, walnut, and cinnamon notes [45,82]. The detection limit for 2-acetylpyrrole is 0.00142 mg/L [82]. 4-methylthiazole imparts a nutty, green flavour [45]. 2-pentylfuran, formed during malting, exhibits a characteristic nutty odour at low concentrations but can become pungent when concentrations are high [83]. Acetals derived from aliphatic aldehydes reacting with alcohols are common in alcoholic beverages, and acetaldehyde diethyl acetal has a nutty aroma [84].

### 2.16. Oxidised

The interaction of dissolved oxygen with other chemical components in beer is known as oxidation, and it is a significant cause of product degradation, leading to a flavour described as oxidised, aged, or stale [85]. Apart from aerating the wort during pitching to improve yeast performance (to ensure sufficient yeast growth and good fermentation performance), brewers should avoid introducing oxygen during mashing, filtration, boiling, and in wort and beer, as it can damage the flavour [86]. The oxidation of fatty acids produces trans-2-nonenal, one of the most significant aldehydes in beer staling, which has descriptors such as cardboard, papery, or cucumber [87]. The flavour threshold for trans-2-nonenal is at 3.0 × 10^−5^ mg/L [68]. Trans-2-nonenal is primarily formed through autoxidation or enzymatic oxidation of unsaturated fatty acids like linoleic acid [68]. The levels of trans-2-nonenal in beer have been found between 1.7 × 10^−4^ and 4.2 × 10^−4^ mg/L [88].

### 2.17. Rotten Eggs

The rotten egg odour can result from bacterial contamination (e.g., *Pectinatus* spp.) or a yeast strain that produces significant amounts of hydrogen sulphide (which causes the rotten egg odour) during fermentation [35]. Hydrogen sulphide has a threshold of 0.005 mg/L [89]. Other volatile sulphur compounds found in beer can also impart a rotten eggs smell, such as methanethiol and ethanethiol, with thresholds of 0.0012 mg/L and 0.0005 mg/L [90]. Besides the rotten egg smell, beers contaminated by bacteria like *Pectinatus* spp. often show heavy sedimentation, haze, and small clots [91]. During yeast fermentation, hydrogen sulphide forms as a by-product of the biosynthesis of sulphur-containing amino acids methionine and cysteine via the sulphate reduction pathway [92].

### 2.18. Skunky

The bitterness of beer is contributed by iso-humulones (iso-α-acids), including iso-n-humulone, iso-cohumulone, and iso-adhumulone [93]. However, during the degradation process, when beer is exposed to sunlight, the iso-α-acids react with sulphur-containing compounds in the presence of riboflavin to produce 3-methyl-2-butene-1-thiol (MBT), which emits skunky, cat-musk, or foxy odours [94,95]. Riboflavin acts as a photosensitiser, and it has been reported that higher concentrations of riboflavin increase MBT formation [94]. MBT has a flavour threshold of 1.0 × 10^−6^ and 7.0 × 10^−6^ mg/L in beer, which is very low for a threshold, making it crucial to understand and control the reaction that creates it [95,96,97]. Both sunlight and conventional fluorescent light bulbs (such as those in supermarkets and other stores) can cause a light-struck reaction; they easily penetrate all glass except dark brown or amber, resulting in the beer developing a skunky aroma [35]. Concentrations of 3-methyl-2-butene-1-thiol were found at levels below 1.0 × 10^−6^ mg/L in beer [97].

### 2.19. Solvent-like

Aliphatic alcohols such as propanol, isobutanol, isoamyl alcohol, and amyl alcohol are the most common higher alcohols found in alcoholic beverages. In addition to contributing to the alcoholic aroma, they can also impart a solvent-like odour to beer and provide a warming sensation on the tongue [34]. When not contributing fruity notes, acetate esters (ethyl acetate, isoamyl acetate) can produce solvent-like odours in the beer aroma when present at higher levels [98]. Ethyl acetate, a prominent ester in beer, is produced in significant quantities during fermentation, mainly when specific yeast strains are used [99,100]. Top-fermenting yeasts produce higher concentrations of esters, including ethyl acetate, compared to bottom-fermenting yeasts because of the higher esterase activity of the yeast strain [101]. When used as a single starter culture, *Pichia kluyveri* and *Wickerhamomyces anomalus* (formerly *Pichia anomalus*) produce high levels of ethyl acetate [102], with *P. kluyveri* also generating large amounts of isoamyl acetate, while *Pichia kudriavzevii* produces high ethyl acetate but low isoamyl acetate [103]. The concentration of ethyl acetate in lager beers has been reported to be up to 31.9 mg/L, with a sensory threshold of 30 mg/L [104].

### 2.20. Sour Milk, Lactic, Acetic

Among the fatty acids, acetic acid can emit sour, vinegary, or pungent odours in beer. It can be produced during and after fermentation through the oxidation of acetaldehyde (an intermediate in alcoholic fermentation) or by acetic acid bacteria in cases of microbiological spoilage [51]. The flavour threshold of acetic acid in beer, where its characteristic vinegary odour becomes detectable, is 130 mg/L [105,106]. Reported sensory thresholds for American lagers range from 71 mg/L to 200 mg/L, with similar values observed in Norwegian lagers and Irish stouts [105,106]. Some of the commonly known acetic acid bacteria are *Acetobacter* and *Gluconobacter*, which are common beer spoilers. However, nowadays, with the implementation of effective cleaning and sanitation procedures in breweries and the effective removal of oxygen, they are no longer considered that important for brewing. Acetic acid bacteria are currently more relevant in cask-conditioned or barrel-aged beers and can cause a visual spoilage effect, such as haziness or ropiness [107]. Lactic acid bacteria (LAB) are beneficial in the mashing process and essential in the production of sour beers, including the Belgian lambic, gueuze, Flanders red, and the German Berliner weisse [108,109]. Unlike traditional beers that use *Saccharomyces cerevisiae,* sour beers often undergo spontaneous fermentations with wild yeasts or mixed-culture fermentations involving genera such as *Lactobacillus* and *Brettanomyces.* Specific strains within these genera contribute to the more acidic and tart flavours [110,111]. The sensory threshold of lactic acid (produced by LAB) in beer varies by style, with values reported at 400–500 mg/L for American lagers and 400 mg/L for Norwegian lagers [105]. Sour beers can be produced through various methods, including spontaneous and non-spontaneous fermentation. These methods can be further divided into sour-malted, sour-mashed, kettle-soured, sour-fermented primary souring, sour-fermented mixed starter, and sour-matured, in which the beer is kept in wooden barrels that harbour wild yeast and bacteria [110,112]. In traditional sour beer production, such as that of lambic beers, no microorganisms are actively added; instead, spontaneous fermentation is preferred [112]. The boiled wort is cooled in open vessels, exposed to the environment, and naturally inoculated by airborne microorganisms before being transferred to wooden barrels that have been previously used for fermentation [113].

### 2.21. Spicy, Clove

Eugenol can emit clove, sweet, floral, rose, fatty, smoky, and fish odours in food products [114]. Its origin may be linked to the use of wild yeasts, microbial contamination, or the oxidation process, with typical concentrations in beer ranging from 0.01 to 0.03 mg/L [80]. The flavour threshold for eugenol in water is between 0.006 and 0.03 mg/L, while in beer it is reported to be 0.04 mg/L [115]. High concentrations of 4-vinyl guaiacol (4-VG) and 4-vinylphenol (4-VP), produced through thermal and enzymatic decarboxylation of ferulic acid and *p*-coumaric acid, respectively, also impart clove and spicy notes. The spicy and clove characters are commonly associated with Bavarian wheat beers and Belgian Witbier [116]. During barrel-aged beer maturation, the choice of wood influences the beer’s chemical and sensory properties. European oak contains significant quantities of eugenol, lactones, and vanillin, whereas acacia has higher levels of guaiacol, 4-vinyl guaiacol, syringol, and flavonoid polyphenols, creating more subtle, spicy notes [117].

### 2.22. Sweaty, Goaty, Rancid, Soapy

The 6- to 12-carbon fatty acids have a distinctive cheesy, goaty, or sweaty flavour, with threshold concentrations of approximately 5 mg/L for caproic acid (hexanoic acid), around 15 mg/L for caprylic acid (octanoic acid), and 10 mg/L for capric acid (decanoic acid) [118]. Concentrations in which these fatty acids are typically found range from 0.1 to 3.83 mg/L for hexanoic acid, 0.04–6.98 mg/L for octanoic acid, and 0.38–2.17 mg/L for decanoic acid [119]. These acids were detected in higher amounts in beers brewed with encapsulated yeast [120]. The capric acid content tends to decrease if the encapsulated yeast is reused for a second batch [121]. However, other aromas, such as papery, grainy, hefeweizen, and spicy, tend to become more dominant instead [121]. Lager yeasts produce higher levels of these fatty acids during fermentation than ale yeasts, leading to caprylic flavours in most lagers [122]. In lambic beers, aroma compounds produced by *Brettanomyces* are characteristic of the style. *Brettanomyces* can produce various fatty acids, such as caprylic and capric acids, along with their ethyl esters [103]. Some authors describe caprylic acid as having a soapy or fatty odour, and it may even be recognised as yeasty by brewers due to its association with specific yeast strains or types [123,124]. Typically refrigerated before brewing, hops can oxidise when kept at room temperature, releasing valeric acid (stinky cheese odour), isovaleric acid (sweaty smell), and caprylic acid (rancid or goaty smell) [125]. Since using aged hops is common practice in gueuze lambic beers, the presence of isovaleric acid is expected. Acetic, lactic, isovaleric, hexanoic, valeric, octanoic, and decanoic acids are all linked with aged hops; consequently, isovaleric acid concentrations in gueuze lambic beers have been reported to range from 1.92 mg/L in Oude Gueuze Vieille to 3.01 mg/L for Cuvee Renée [126].

### 2.23. Yeasty, Meaty

Residual yeast left after fermentation or in packaged beer can produce a yeasty flavour that may develop into marmite or meaty notes over time. To avoid these undesirable flavours, yeast should be removed after primary fermentation and the diacetyl rest. If beer conditioning occurs in bottles or casks with yeast, the yeast count should be maintained at an appropriate level [127]. MHF (5-Methyl-4-hydroxy-3(2H)-furanone), known for its meaty or brothy smell, is usually present in beer at levels of 0.12–0.86 mg/L, which is far below its flavour threshold in water of 8.3 mg/L [128]. MHF forms during the Maillard reaction during the kilning of green malt and during mashing, boiling, and fermentation [128]. Flavours like yeasty and meaty are often associated with cloudy wheat beers, which are unfiltered and allow yeast to contribute to their distinctive flavour [129].

## 3. Strategies for Controlling Off-Flavours That Appear in Beer During Manufacturing and Storage

Many off-flavours can cause issues during brewing and negatively affect the final product. The objective was to create a troubleshooting guide that includes the most relevant odours and offers brewers causes and solutions for common flavour problems. The guide was developed by analysing the latest research in the brewing industry and includes information on the odour descriptor, chemical compounds, origin, and corrective actions. It is also important to note that certain flavours are desirable in specific beer styles, while in other cases, they are considered off-flavours (Table 1).

**Table 1:** Troubleshooting guide for odours that appear in beer.

No.	Descriptor	Compound/Source	Beer Styles Commonly Associated	Origin	Corrective or Preventive Actions	Reference
1.	Apple	Acetaldehyde	Young beers (any style), lagers, Bavarian wheat beers	Stressed or incomplete fermentation.	Proper yeast management involves ensuring the correct concentration of healthy yeast is pitched, within the range of 26 to 35 × 10^6^ cells/mL.	[21,56,130,131,132,133,134,135,136,137,138]
				Insufficient oxygen supply during the yeast’s growth phase can lead to higher acetaldehyde levels.	Wort aeration with oxygen to maintain cell viability (7–12 mg O_2_/L).	
				Pitching rates of yeast and wort composition.	Ensure a wort zinc concentration range from 0.48 to 1.07 mg/L.	
				Zinc deficiencies in wort can lead to excess acetaldehyde production.	Provide adequate Free Amino Nitrogen (FAN) concentrations in the wort to support yeast growth and fermentation (100–140 mg/L in normal gravity 10–12 °P wort).	
				Leaving non-viable yeast in maturation for extended periods of time.	Concentrations of acetaldehyde can be lowered by prolonged maturation with healthy yeast	
				Microbial contamination of beer (*Zymomonas* or *Acetobacter*).	Proper sanitation to avoid bacterial infection.	
				Use of adjunct sugars.	Avoid the use of adjuncts with high glucose concentrations, which will inhibit the fermentation rate and the yeast growth.	
2.	Alcoholic	Ethanol, Isoamyl alcohol, Active-amyl alcohol, n-Propanol, Isobutanol, Butanol	High-gravity ales, Belgian ales (e.g., Tripel, Dubbel), barleywine, Imperial stout, strong lagers	High fermentation temperature.	Lower fermentation temperatures and higher pressure to reduce the extent of higher alcohol production.	[22,24,137,139]
				Addition of higher amounts of fermentable sugars in the wort (e.g., glucose syrup).	Avoid adding large amounts of fermentable sugars, which will expose the yeast to various stresses.	
				Selection of yeast strain.	An adequate concentration in the range of 130–150 mg/L of FAN in wort to support optimum yeast growth and fermentation efficiency.	
				Low content of FAN to support optimal yeast growth.		
				Oxygenation and agitation during fermentation.		
				Lower pressures during fermentation encourage yeast growth.		
3.	Boiled potato	Methional	Any aged beers	Developed during wort boiling.	Selection of malt: a higher proteolytic malt modification will transcribe into an increase in free and bound aldehyde content at the end of wort boiling.	[20,140,141]
				Insufficient yeast reducing activity (due to weak or stressed metabolism).	During fermentation, if the yeast metabolism is optimal, then there is a reducing activity which will transform methional to the corresponding alcohol.	
					During storage, the methional can also be reduced, and the presence of oxygen causes a minor reduction.	
4.	Buttery	Diacetyl	Any beer styles	Any of the precursors that stay in the beer after yeast removal is liable to be converted.	Worts with a lower FAN content produce less diacetyl (but worts with FAN values < 122 mg/L will trigger the opposite response) as α-acetolactate production is related to valine metabolism.	[32,34,35,142,143]
				Low values for valine in wort.	Valine supplementation (100 to 300 mg/L) can decrease the diacetyl concentrations.	
				Short times for the secondary fermentation.	Yeast cells possess the enzymes required to assimilate and reduce diacetyl. Yeast should not be stressed or rushed, also, a slight temperature increase towards the end of fermentation can help with the diacetyl clean up.	
					Diacetyl will be reduced through a regular maturation below threshold values in up to 15 days.	
5.	Caramel, Malty	Furans and furan derivatives. Heterocycles and pyrazines. Carbonyl compounds and related Maillard reaction products.	Ales (Belgian, English, German), stout, porters, barleywines, Munich dunkel, Weizen bock, Helles, bock, amber lager, dark lager	Compounds are developed during the malt kilning and the wort boiling.	The strategic selection of malt is crucial: caramel malt provides enhanced caramel notes, while roasted malt contributes more roasted or malty flavours. Caramel malts are produced with an additional step before kilning called stewing, where the high moisture content of steeped grains is combined with a temperature of 60 °C. This process leads to saccharification, followed by increasing the temperature to 120 °C for caramelisation, and finally to 180 °C for pyrolysis in a roasting drum. Roasted malts are produced similarly to pilsner malts, except the final kilning temperature is raised to around 200–220 °C.	[37,38,39,144,145]
				Beer storage and ageing.	Adjusting malting parameters such as time and temperature during kilning will alter the malt’s composition. The presence of sulphur compounds inhibits the Maillard reaction, and sulphur dioxide reduces the colour of the malt.	
					Controlling the brewing steps: wort boiling time, temperature, pH, and the amino acids and sugars present, will influence the volatile compound formation.	
					Ageing and storage can alter the beer: duration, temperature and oxygen exposure can create new caramel and aroma notes.	
					Furfural is formed in considerable amounts during mashing and hopping steps, but almost all is reduced by yeast during fermentation; is formed again during storage and ageing.	
6.	Cidery	Acetaldehydes, Ethyl esters	Lambic, Gueuze	Choice of yeast strain.	Acetaldehydes can be prevented by aeration of the wort before adding yeast.	[145,146,147]
				Weak or incomplete fermentation.	Avoiding exposure to oxygen during fermentation	
				Composition of the fermentation medium and fermentation conditions.	Avoid bottling or kegging the beer too early.	
7.	Cooked vegetable	Dimethyl sulphide	Any beer styles	Precursor SMM is produced during the grain germination, in the seed germ and rootlets and is converted during malt kilning and wort boiling.	To reduce SMM levels, consider replacing malt with adjuncts, the use of rootlet inhibitors, increasing the intensity of kilning and choosing a 2-row variety instead of a 6-row.	[46,47,48]
				Precursor DMSO also originates from malt but is reduced to DMS by yeast during fermentation.	Intensive wort boiling will reduce the DMS concentration.	
				DMSO reduction into DMS is elevated at low temperatures, high pitching wort pH, low free amino nitrogen levels and high original gravity.	Location of the brewery can affect the conversion of SMM; a wort that boils at a lower temperature in an area with increased elevation will have significantly less conversion of SMM into DMS in the kettle.	
					The amount of DMS produced by yeasts varies between strains.	
					DMS produced during fermentation can be eliminated by CO_2_ stripping.	
					At higher fermentation temperatures, the diffusion rate of CO_2_ is increased, which helps to eliminate DMS from green beer.	
					*Saccharomyces pastorianus* var. *carlbergensis* reduces less DMSO than *Saccharomyces cerevisiae*.	
8.	Estery, fruity	Isoamyl acetate, ethyl hexanoate, ethyl octanoate, phenylethyl acetate, 3-sulfanyl-4-methyl-pentan-1-ol, 4-methyl-4-sulfanyl-2-pentanone, 3-sulfanylhexan-1-ol, γ-lactones, γ-decalactone, oak-lactone.	English ales (English Bitter, Pale Ale, Mild Ale), Belgian ales (Dubbel, Tripel, Saison), Wheat beers (Hefeweizen, Weizen bock), sour beers, wild ales	Esters are synthesised during fermentation, and the amount depends on the yeast strain and the fermentation temperature.	Selection of yeast, a greater retention of esters inside the cells is characteristic of lager yeast strains.	[35,42,145,148]
				Ester production increases when there is a high aeration, a high amount of nutrients and low pitching rates for yeast.	Lower fermentation temperature slows down yeast metabolism and reduces ester synthesis.	
				High-gravity worts or high sugar concentrations promotes ester formation.	Avoiding overstressing or underpitching the yeast to have lower concentration of esters.	
				Lactones originate from malt, hops and yeast metabolism, and oak-lactone is mainly present in beers aged in oak casks.	Different varieties of hops give a particular flavour profile.	
9.	Grassy	Hexanal	Any Beer Styles (Especially pale lagers)	Use of large quantities of hops that are fresh, poorly stored, or not properly dried.	Proper storage techniques for raw materials and beer.	[9,35]
				Improper storage of malt.	Selection of hops.	
					Yeast can remove aldehydes by reduction to their saturated alcohol counterparts.	
10.	Hoppy	Linalool, geraniol, β-damascenone, β-citronellol, esters, organic acids, myrcene, α-humulene, β-caryophyllene and β-farnesene.	German Pils, Czech Pilsners, Kölsch, American pale ale, India pale ale	Early kettle addition causes extensive loss of the volatiles by evaporation during boiling.	Late hopping and dry-hopping additions preserve polar oxygenated terpenoids (e.g., humulene epoxides or linalool oxides).	[57,61,145]
				Dry hopping at warmer temperatures (around 20 °C) increases the extraction of myrcene.	Dry hop at cool temperatures (10–14 °C).	
				Long dry hop duration (more than 3 days) increases the extraction of harsh, green flavours.	To avoid astringency and a green, harsh character, keep the contact time shorter, 1–3 days.	
				Oxygen exposure during dry hopping oxidises hop oils.	Add hops to the tank while running CO_2_ into it and avoid introducing oxygen.	
					Agitate hops to improve extraction.	
11.	Husky, grainy	Isobutyraldehyde, ethyl nicotinate, *o*-aminoacetophenone	Pale lager, cream ales, wheat beers, Belgian ales, German pils, Czech pale lager	Use of freshly made malt.	Use of malt that has gone through an appropriate rest phase.	[43,63,145]
				Isobutyraldehyde can appear if malt has been crushed too finely, mashed for too long, sparged with water at too hot a temperature or oversparged.	Avoid crushing malt too finely, mashing for too long, overheating or oversparging.	
				Ethyl nicotinate formation is a spontaneous chemical condensation reaction between nicotinic acid and ethanol during beer ageing.	Storing beer at low temperatures.	
12.	Medicinal	Chlorophenols, bromophenol	Any beer styles	Production of chlorophenols from chlorination.	Thoroughly drain and rinse equipment to remove chlorine-based cleaning agents.	[69,149]
				Inadvertent steam leaks (through heat exchangers) into product steam or hot liquor supplies.	Use sulphite systems instead of chlorine-based ones if possible.	
				Condensate residues left after steam sterilisation of mains, vessels, kegs, and casks.	Prevent steam leaks and ensure proper maintenance of heat exchangers.	
				Concentration of chlorophenolic compounds in boiler feed water.	Control and monitor brewing water quality to avoid build-up of contamination.	
				In situ generation from active chlorine residues or boiler feed additives.	Avoid continued use of chlorine and chloramine in the brewing water. Tap water needs to be filtered, boiled, or left to stand for some time to eliminate free chlorine, which is typically found at 0.05–0.25 mg/L.	
					Yeast strains with low phenol production should be selected, and the use of wild yeast should be avoided. Additionally, malt should not be ground too finely to minimise the release of phenolic compounds.	
13.	Metallic	Iron (Fe^2+^), Copper	Any beer styles	Contamination from brewery equipment and containers (e.g., pipes, tanks, kegs, cans) used during fermentation, conditioning, filtration, carbonation, and storage.	Avoid fittings, containers, vessels, tanks, and sealants that can corrode.	[52,70,72]
				Poor plumbing system in the municipality or brewery.	Use food-grade, stainless steel containers.	
				Copper can enter the water if the pH of the water is acidic, and the pipes are made of copper.	Most of the iron entering the mash is retained and subsequently removed with the spent grains during wort filtration.	
					Pilsner malt produces a sweet wort with a lower iron concentration than wort made with roasted malts.	
14.	Mouldy	geosmine, 2-methyl isoborneol, 1-octen-3-one, chloranisoles, TCA, 2-Ethyl-fenchol	Any beer styles	Malting equipment contaminated by microorganisms. Some dormant microorganisms are activated during steeping, and germination provides high levels of moisture and solubilised or partially solubilised nutrients.	Proper cleaning and sanitisation of malting and brewing equipment.	[73,74,76,150]
				TCA develops on the premises of the brewery, or it comes from the packaging materials.	Good storage conditions for the malt, the moisture content of the malt kept should be kept at around 4.5%.	
					Preventive measures include optimising carbon filtration of source water, improving cleaning-in-place procedures, packaging processes, and raw material storage, and upgrading the brewery’s ventilation and pasteurisation systems.	
15.	Nutty	2-acetylfuran, 2-Pentylfuran, 2-acetylpyrrole, 4-methylthiazole	Mild ale, brown ale, Scottish ales, English porters, Vienna lager, Märzen, bocks	Pyrroles, pyrazines, and thiazoles are all formed during the kilning due to the Maillard reaction.	The Maillard reaction occurs in temperatures above 50 °C.	[45,82,145]
				2-acetylpyrrole appears during the roasting of the malt.	2-acetylpyrrole decreases with a higher roasting temperature.	
				Maillard reaction products are also formed during mashing and wort boiling.	The Maillard products depend on the level of malt modification and ratio of nitrogen, carbohydrates, and sulphur (e.g., the incorporation of sulphur forms thiazoles).	
16.	Oxidised	*trans*-2-nonenal	Any beer styles (especially in pale lagers and aged beers)	Spring barley contains higher levels of polyunsaturated fatty acids.	The crop year, barley variety, and the application of a zinc-containing fertiliser can affect the antioxidant content that suppresses aldehyde formation.	[68,87]
				Use of malt with a high lipoxygenase activity.	Winter barley exhibits higher antioxidant activity because it has a thicker husk.	
				High temperatures, elevated oxygen levels and the presence of pro-oxidants, such as transition metal ions (copper and iron), accelerate the rate of autoxidation.	During germination recirculate air enriched with CO_2_ to suppress oxidation.	
17.	Rotten Eggs	Hydrogen Sulphide	Most common in lagers and young beers	Hydrogen sulphide can be produced by certain brewing yeast strains during fermentation.	Picking a yeast strain with a reduced production of hydrogen sulphide. Lager yeast will produce more sulphur compounds, but the smell will also dissipate after fermentation.	[35,73,91,151,152,153]
				Use of sugar syrups in wort production decreases the malt ratio, thereby reducing the nitrogen content of the wort.	Avoid the nitrogen starvation of bottom-fermenting yeasts during the exponential growth as this will produce more hydrogen sulphide.	
				*Pectinatus* are anaerobic bacteria that can contaminate packaged beer and produce hydrogen sulphide.	*Pectinatus* spp. appear to only tolerate beers with ethanol up to 4.4% (*w*/*v*) and a pH of 4.0–4.5.	
				*Pectinatus* is usually isolated from non-pasteurised, packaged beer that has lower alcohol levels and slightly higher pH.	Improved brewery hygiene and increased vigilance for spoilage bacteria are highly important.	
				Storage conditions of the packed product.	*Pectinatus* spp. grows between 15 and 40 °C, with an optimum at 32 °C.	
18.	Skunky	3-methyl-2-butene-1-thiol (MBT)	Any beer styles	Exposure of beer to UV lights (photooxidation) causes a reaction between the light, riboflavin in the beer and the hop-derived iso-α-acids.	Sunstruck flavours occur especially when the beer bottle is translucent or green, and without UV protection, the colour for the best protection is brown.	[95,154]
					Hop polyphenol and tannin extracts have been shown to enhance beer’s light and storage stability by inhibiting oxidative and light-induced off-flavours.	
					Compounds such as tryptophan, ascorbic acid, and tryptophol can act as off-flavour inhibitors by scavenging radicals and reducing the formation of light-struck compounds.	
19.	Solvent-like	Ethyl acetate Fusel alcohols (see row no. 2 for origin and corrective actions)	High-gravity beers, British strong ale, Imperial stout, barleywine	Selection of the strain of yeast.	Top fermenting strains generally produce more esters and higher alcohols than bottom fermenting strains.	[100,145,146,155,156]
				Composition of the fermentation medium.	Fermentation temperature should be reduced (the highest content of ethyl acetate is produced at 20 °C).	
				Fermentation conditions.	Ensure adequate levels of unsaturated fatty acids in wort, as they regulate acetate ester production.	
					Control the carbon-to-nitrogen ratio in the fermentation medium (increased carbon or nitrogen content is correlated to higher acetate ester production).	
					Reduce wort gravity to lower acetate ester formation (high-gravity brewing increases acetate ester production).	
					Increase wort aeration to decrease ester concentration.	
20.	Sour milk, lactic, acetic	Lactic acid Acetic acid	Sour beers, American wild ale, mixed-fermentation sour beers, Berliner weisse gose, lambic, gueuze, Flanders red ale, oud bruin	Lactic Acid Contamination through ingredients, equipment, storage, and packaging.	Practice proper hygiene and sanitation practices to prevent contamination by lactic acid bacteria in ingredients, equipment, storage, and packaging.	[107,112,145,157,158]
				Acetic Acid Contamination.	Practice proper sanitation to control microbial contamination by *Acetobacter* or *Glucanobacter* in wort, beer dispensing systems or cask-conditioned ales.	
				Wild Yeast Contamination.	Practice proper sanitation and proper yeast management to prevent wild yeast growth.	
				Exposure to Oxygen.	Minimise headspace in fermenters to reduce oxygen contact. Ensure proper CO_2_ or nitrogen purging of tanks, hoses, pipes, and kegs during all stages (fermentation, filtration, packaging).	
				Fermentation Conditions.	Maintain controlled fermentation conditions to prevent contamination and ensure quality.	
				Insufficient Pasteurisation.	Pasteurise the beer at a minimum of 15 pasteurisation units (1 PU = 1 min at 60 °C to reduce microbial contamination.	
				Storage and Packaging.	Ensure proper storage and packaging by preventing exposure to oxygen, controlling temperature, and using sealed and sanitised containers.	
21.	Spicy, clove	Eugenol 4-vinyl guaiacol 4-vinylphenol	Hefeweizen, dunkles Weissbier, Weizenbock, saison, Belgian Witbier, Belgian Dubbel, Belgian dark strong ale, Belgian tripel, lambic, gueuze	Microbial contamination.	Implement an efficient CIP (clean-in-place) system to prevent contamination.	[80,116,145]
				Oxidation product in aged beer.	Minimise oxygen exposure during packaging and storage to prevent oxidation.	
				Wild yeast infection (e.g., *Saccharomyces diastaticus*).	Practice good sanitation to avoid a wild yeast infection.	
				Malted and unmalted wheat.	Avoid using malted and unmalted wheat, as they contribute to 4-VG and 4-VP formation.	
				Ferulic Acid Rest for 4-VG.	Avoid performing a ferulic acid rest during mashing, as it increases 4-VG production by releasing additional free ferulic acid into wort.	
22.	Sweaty, goaty, rancid, soapy	Caproic acid Caprylic acid Capric acid Valeric acid Isovaleric acid	Mixed-fermentation sours, lambic, gueuze, Brett beers	Microbial contamination with *Brettanomyces.*	Practice good sanitation practices to prevent contamination.	[103,121,123,125,126]
				By-product of the yeast metabolism.	Select an appropriate strain and type of yeast to minimise undesired by-products.	
				Use of encapsulated yeast.	Use free yeast instead of encapsulated yeast to control yeast metabolism better.	
				The use of old, oxidised hops.	Use fresh hops that are properly stored to avoid oxidation and off-flavours.	
23.	Yeasty, meaty	Yeast 5-Methyl-4-hydroxy-3(2H)-furanone (MHF)	Any beer styles (most likely in bottle-conditioned or cask conditioned beers)	Yeast autolysis.	Check yeast count (0.5–2 × 10^6^ cells/mL) for cask and bottle-conditioned beer.	[127,128]
				Strain of yeast.	Limit the time the beer spends on yeast in the tank.	
				Prolonged contact with the yeast.	Ensure pitched yeast has >90% viability.	
				For MHF, the type of malt used.	Select the appropriate yeast strain for the fermentation process.	
					Limit the time beer spends on yeast to prevent autolysis.	
					To avoid MHFs, use lager and ale malts, as they have no furanones.	
					Avoid using stewed malt kilned at elevated temperatures, as they have the highest levels of furanones.	

## 4. Conclusions

This review offers a detailed analysis of beer aroma, a complex mix of volatile compounds. Identifying key odourants and the mechanism behind their formation is essential for maintaining product quality and consumer satisfaction. Addressing common issues such as oxidation, microbial contamination, or chemical instability involves implementing both preventative and corrective strategies within beer production. Ongoing progress in flavour research is helping brewers and researchers in better identifying and controlling aroma-active compounds.

## Data Availability

No new data were created or analyzed in this study.

## Abbreviations

The following abbreviations are used in this manuscript:

OAV	Odour activity value
HDMF	4-hydroxy-2,5-dimethylfuran-3(2H)-one
DMS	Dimethyl sulphide
SMM	s-methylmethionine
DMSO	Dimethyl sulfoxide
TCP	trichlorophenol
MBT	3-methyl-2 butene-1-thiol
LAB	Lactic acid bacteria
4-VG	4-vinyl guaiacol
4-VP	4-vinylphenol
MHF	5-methyl-4-hydroxy-3(2H)-furanone
FAN	Free amino nitrogen
